# The Role of Patient Motivation in Single-Sided Deafness: Patterns in Treatment Selection and Cochlear Implant Outcomes

**DOI:** 10.3390/jcm14248944

**Published:** 2025-12-18

**Authors:** Leena Asfour, Allison Oliva, Erin Williams, Meredith A. Holcomb

**Affiliations:** Department of Otolaryngology, University of Miami, Miami, FL 33136, USA

**Keywords:** SSD, cochlear implant, treatment, motivation, single sided deafness, telehealth, cochlear implantation, treatment selection

## Abstract

**Background/Objectives:** Single-sided deafness (SSD) treatment options include Contralateral Routing of Signal (CROS) or Bilateral Routing of Signal (BiCROS) systems, bone conduction devices, cochlear implants (CIs) and no intervention. Aligning treatment recommendations with patient motivations is fundamental for satisfaction and successful outcomes. At our institution, a structured telehealth consultation precedes formal testing and includes treatment motivation exploration and comprehensive review of all interventions. This study examined SSD treatment motivations and their association with pursuing cochlear implantation. **Methods:** Adults who completed a pre-treatment SSD telehealth consultation over a four-year period were identified. Charts were retrospectively reviewed for demographics, SSD characteristics, treatment motivations, treatment choice, and CI outcomes. **Results:** A total of 122 adults were evaluated. Mean age was 56.3 (±13.0) years, and 59.8% were male. Mean SSD duration was 10.8 (±15.8) years. The most common etiology was sudden sensorineural hearing loss. The top primary motivations were improving overall hearing (23.0%), restoring hearing to the deaf ear (22.1%), and improving hearing in noise (21.3%). Most patients (45.1%) opted for a hearing aid, CROS or BiCROS system; 38.5% chose CI; and 14.8% declined treatment. Only 57.4% of those who selected CI had the implant, primarily due to surgery avoidance (31.5%) and insurance limitations (10.5%). Motivation did not predict treatment choice or CI receipt. Among CI recipients (*n* = 27), those motivated by hearing restoration demonstrated poorer speech outcomes and datalogging. **Conclusions:** Improving overall hearing and restoring hearing to the deaf ear were the most common motivations for seeking SSD treatment. Adult CI recipients had similar motivations to those who chose non-surgical options.

## 1. Introduction

Approximately 0.14% of adults, or about 350,000 people, in the United States (US) experience single-sided deafness (SSD) [1]. SSD is defined as normal or near-normal hearing in one ear with complete or severe hearing loss in the other ear. This condition can significantly impair a person’s ability to localize sound or understand speech in noisy environments as benefits of binaural hearing like summation, interaural time differences, and the head shadow are absent [2,3,4].

SSD in adults arises from a variety of congenital and acquired etiologies. According to the American Cochlear Implant Alliance guidelines, the most common acquired SSD in adults is sudden or progressive idiopathic sensorineural hearing loss, which accounts for over 50% of adult SSD cases [5,6]. Other contributing factors include age-related hearing loss (presbycusis), ototoxic drug exposure, and genetic mutations [5,6]. Symptoms associated with SSD include tinnitus, hyperacusis, aural fullness, and vestibular changes, all of which can range from non-bothersome to debilitating [5,6]. Beyond physical conditions, SSD carries emotional and psychosocial consequences. Patients with acquired hearing loss often struggle with denial and grief following the hearing loss diagnosis [7]. Additionally, SSD can lead to social withdrawal, frustration, anxiety, feelings of isolation, and fear of losing hearing in the contralateral ear [8].

In evaluating patients with SSD, our institution adheres to a protocol aimed at providing comprehensive counseling and testing to assist patients with treatment selection. Central to this protocol is a pre-treatment telehealth visit facilitated by experienced audiologists, wherein patients are asked about their primary and secondary motivations for seeking SSD treatment and then meticulously educated about various interventions, including a bone conduction device (BCD), a CROS (Contralateral Routing of Signal) system, and a cochlear implant (CI). BCDs work by transmitting sound from the poorer-hearing ear through the bone of the skull to the inner ear on the unaffected side [9]. CROS systems use a microphone placed on the impaired ear to transmit sound to a receiver on the better-hearing ear [7]. CIs are surgically implanted devices that directly stimulate the cochlear nerve, providing sound perception in the ear with severe or complete hearing loss [10]. Finally, patients are informed that they may also choose to forgo intervention and continue living with their current hearing status. When choosing an SSD treatment option, adult patients typically consider practical factors like comfort and aesthetics, functional outcomes of the devices, and how interventions will integrate into their lifestyle [7]. As such, personalized counseling ensures that patients receive tailored recommendations based on their unique hearing profiles and preferences, fostering informed decision-making and optimal treatment outcomes [5,6].

Understanding patient motivations is particularly important for CI clinicians, as each treatment option carries unique benefits and limitations. For example, rerouting systems like BCDs and CROS systems can improve speech in noise but do not restore sound localization [11,12,13,14] or alleviate tinnitus. In contrast, cochlear implantation has demonstrated significant benefits across multiple domains, including single-word recognition [15,16,17,18,19,20], sentence understanding in noise [15,16,17,20,21], tinnitus reduction [15,18,21,22,23,24], sound localization [15,21,25,26,27], and hearing-related quality of life [15,17,19,21,28,29,30]. Moreover, commitment to consistent CI use is critical for achieving optimal outcomes, as increased CI wear time is associated with greater improvements in speech understanding and quality of life [19].

Despite the importance of patient-centered counseling [6], there is currently no research quantifying the primary and secondary goals of patients undergoing SSD treatment. While a 2021 study by Marx and colleagues reviewed treatment choice in a sample of 155 patients with SSD, their study did not address *motivation* for patients’ treatment modality selection [31]. This gap in the literature highlights the need to better understand how patient goals influence decision-making and outcomes for the SSD population.

The primary aim of this study was to explore patient treatment motivations and identify whether SSD patients with specific treatment goals choose cochlear implantation more frequently. A second goal was to determine if various treatment motivations for cochlear implantation were associated with differences in CI wear time and/or speech outcomes. Results from this study can help guide counseling on treatment options for future SSD patients.

## 2. Materials and Methods

### 2.1. Data Collection

Patients who underwent a pre-treatment SSD telehealth evaluation in the University of Miami’s Hearing Implant Program [Miami, FL, USA] between 1 January 2021 to 31 December 2024 were identified from the electronic medical records of a tertiary referral center. All adult patients (aged ≥18 years) with a diagnosis of SSD and who completed the telehealth appointment were included. Patient charts were reviewed for demographic information, etiology and duration of deafness, audiologic metrics, and primary and secondary goals of SSD treatment.

The telehealth counseling visit was administered by a licensed audiologist in the Hearing Implant Program to educate patients on SSD and treatment options. To ensure consistent messaging and enhance patient understanding of each treatment option, our audiology team utilizes a shared slide deck during the pre-treatment telehealth appointment. This standardized resource includes easy-to-understand language and images of SSD interventions and structured questions aimed at discerning each patient’s primary motivation for pursuing treatment. Patients were asked to review a list of possible treatment motivations and identify their primary and secondary motivations for seeking SSD treatment, and this was recorded in the chart note. During chart reviews, “unknown” was used to delineate those whose chart did not list their motivation. The patient’s treatment choice at the end of the visit was also collected, and treatment choices were categorized into five groups: (1) BCD, (2) CROS/BiCROS/HA, (3) CI, (4) no treatment, or (5) undecided. Reasons for not pursuing a CI were also collected.

For patients who underwent cochlear implantation, datalogging time (average hours per day) and word recognition scores (WRSs) at 12 months post-CI were collected. The Consonant–Nucleus–Consonant (CNC) monosyllabic word test [32] was used for English-speaking adults, and the Auditec Spanish Bisyllable [33] word lists were used for Spanish-speaking adults. CI WRSs were listed as percent words correct, and recorded material was administered for all patients.

### 2.2. Statistical Analysis

All analyses were conducted using SPSS (IBM SPSS Statistics, Armonk, NY, USA, Version 29.0.2.0) and R (R version 4.0.1). Descriptive statistics were first computed for the demographic, SSD characteristics, treatment motivation, and treatment choice data. Pearson’s Chi-Square test was used to determine if there was an association between initial treatment choice and primary treatment motivation.

A Fisher–Freeman–Halton exact test was used to determine if there was an association between primary motivation for SSD treatment and receipt of cochlear implantation. Two-sample *t*-tests were used to determine if there were significant differences in datalogging time and word recognition scores at 12 months based on primary treatment motivation.

## 3. Results

### 3.1. Patient Characteristics

Over the course of 4 years, 122 adult patients underwent an SSD telehealth evaluation at our institution and were included in the study. Patient characteristics are summarized in Table 1. Mean age was 56.7 (SD = 12.7; range 23–89), and 59.8% were male. Most were white (84.4%) and about half were non-Hispanic (49.2%) vs. Hispanic (42.6%). English was the most common primary language (77.9%), followed by Spanish (20.5%). About three quarters of the patients (74.6%) had private insurance, with most of the remaining being insured by a Medicare advantage plan (18.9%) and Medicaid (5.7%).

**Figure 1:**
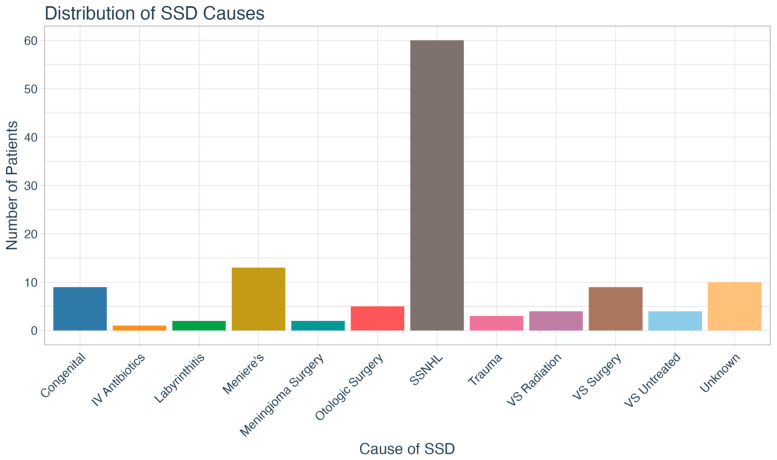
Distribution of single-sided deafness causes. SSD = single-sided deafness; IV = intravenous; SSNHL = sudden sensorineural hearing loss; VS = vestibular schwannoma.

The SSD audiometric characteristics of our patient sample are shown in Table 2. The mean unaided WRSs of the better and worse ears were 97.5% (SD = 6.7) and 6.8% (SD = 12.1), respectively. The mean three-frequency (500, 1000, 2000 Hz) pure tone averages (PTAs) of the better and worse ears were 26.0 (SD = 12.3) dB and 90.8 dB (SD = 21.69), respectively. The average time with SSD was 10.8 (SD = 5.8) years, and the most common cause was sudden sensorineural hearing loss (SSNHL), as shown in Figure 1.

### 3.2. Primary and Secondary Treatment Motivations

The three most common primary treatment motivations included improving overall hearing (*n* = 28; 23.0%), restoring hearing to the deaf side (*n* = 27; 22.1%), and improving hearing in noise (*n* = 26; 21.3%).

**Figure 2:**
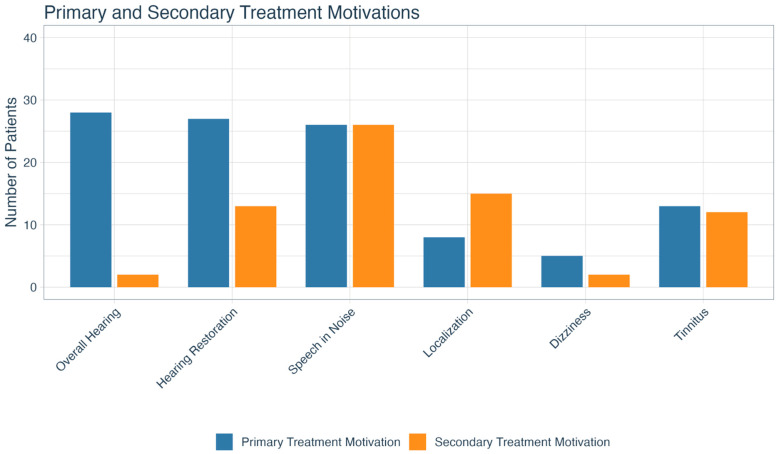
Primary and secondary treatment motivations for patients with SSD. SSD = single-sided deafness.

The three most commonly reported secondary motivations were improving hearing in noise (*n* = 26; 21.3%), localization (*n* = 15; 12.3%), and restoring hearing to the deaf side (*n* = 13; 10.7%). A secondary motivation was not listed for almost half (*n* = 52; 42.6%) of the cohort. The breakdown of all primary and secondary treatment motivations is shown in Figure 2.

### 3.3. Initial Treatment Choice

**Figure 3:**
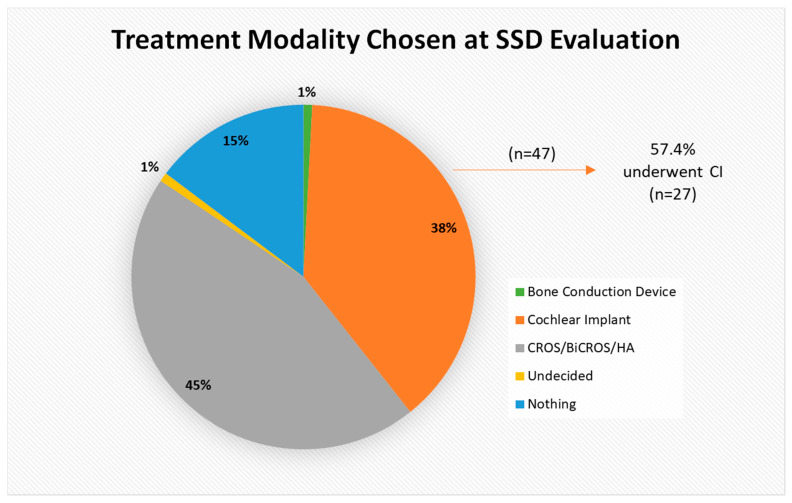
Breakdown of treatment modality chosen at SSD evaluation. CROS = contralateral routing of sound; BiCROS = bilateral contralateral routing of sound; HA = hearing aid.

**Figure 4:**
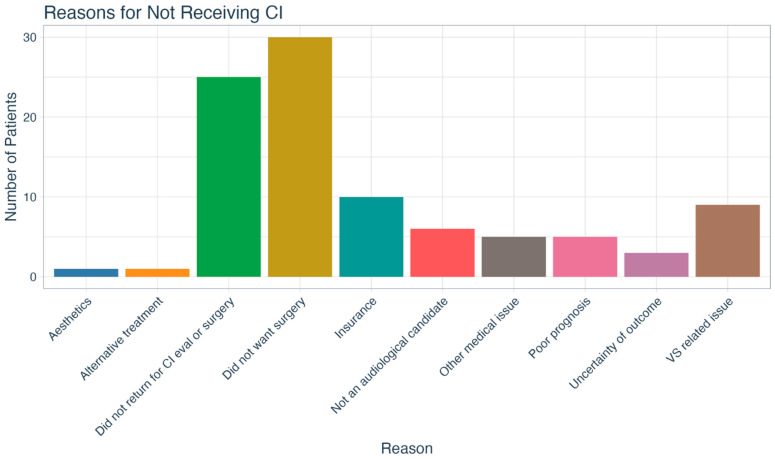
Reasons cited by patients for not pursuing cochlear implantation. CI = cochlear implant; VS = vestibular schwannoma.

Our patient sample’s initial treatment choices are shown in Figure 3. Nearly half of patients (*n* = 55; 45.1%) opted for the hearing aid (HA) option and 14.8% (*n* = 18) chose no treatment. Thirty-eight percent (*n* = 47) opted for a CI as their initial treatment choice, but only 57.4% (*n* = 27) of those underwent implantation. The most common reason patients cited for declining a CI was a desire to avoid surgery (*n* = 30; 31.5%), though in some cases the reason was related to insurance coverage (*n* = 10; 10.5%). Furthermore, 26.3% (*n* = 25) did not follow through with the CI evaluation and/or surgery even though they opted to proceed with CI as a treatment option in the telehealth appointment. Other reasons for not pursuing CI are delineated in Figure 4.

**Figure 5:**
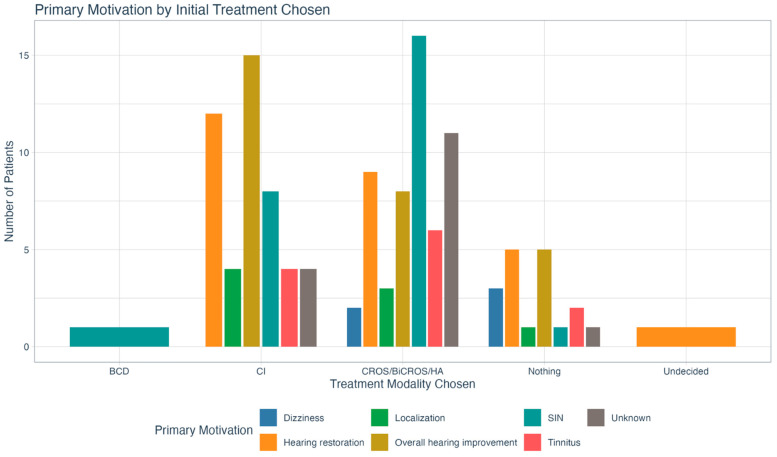
Delineation of initial treatment choice by primary treatment motivation. There was no statistically significant relationship between the two variables. BCD = bone conduction device; CI = cochlear implant; CROS = contralateral routing of sound; BiCROS = bilateral contralateral routing of sound; HA = hearing aid; SIN = speech in noise.

Figure 5 outlines primary treatment motivations by initial treatment choice. Pearson’s Chi-Square testing did not show any significant relationship between the two variables (χ^2^ (24) = 29.134, *p* = 0.215).

### 3.4. Cochlear Implant Recipients

Of patients who proceeded with cochlear implantation (*n* = 27; 22.1%), the most common primary motivations for selecting that treatment option included wanting to improve overall hearing (*n* = 8; 29.6%) and to restore hearing to the deaf side (*n* = 7; 25.9%). A Fisher–Freeman–Halton exact test showed no significant association between primary motivation for SSD treatment and receipt of CI (*p* = 0.197).

**Figure 6:**
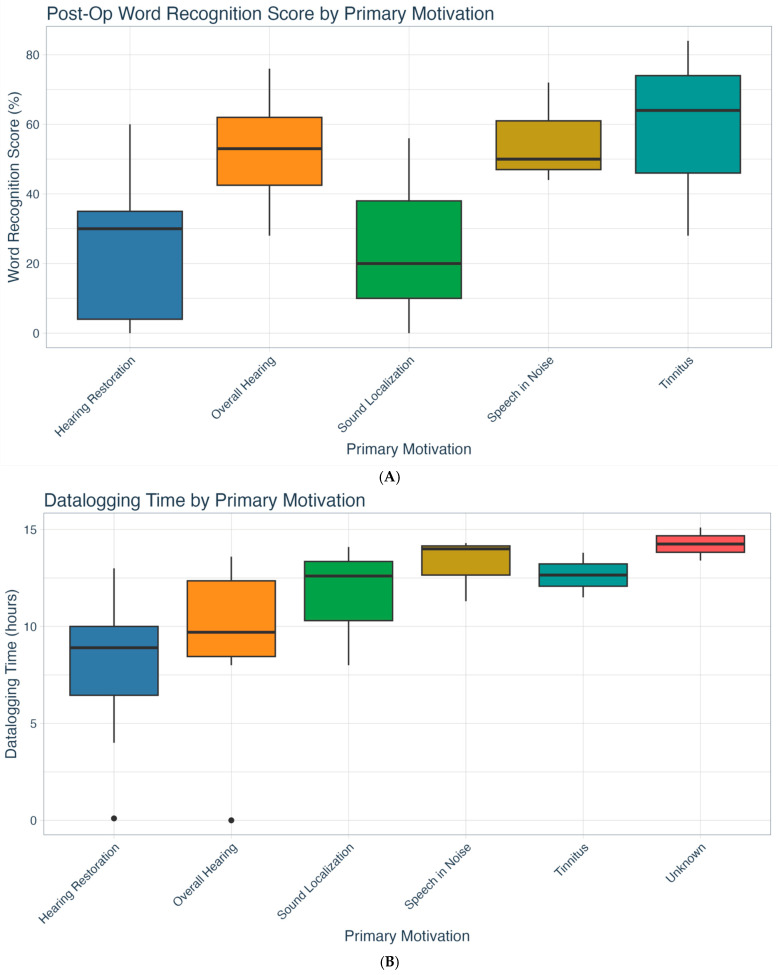
(**A**) One-year post-op CI word recognition scores by treatment motivation (*n* = 23). (**B**) CI datalogging time by primary treatment motivation (*n* = 24). CI = cochlear implant.

CI WRSs during the 1-year post-operative test interval were available for 23 of the patients. The mean overall WRS was 43.1% (SD = 26.7; range 0–92). Figure 6A shows the WRSs by primary treatment motivation. Patients with a primary treatment motivation of restoring hearing to their deaf ear had substantially lower mean WRSs (24.0 ± 22.5) compared to the remainder of the group (51.5 ± 24.5). The difference was statistically significant (*t*(21) = −2.53, *p* = 0.019), with a mean difference of −27.5 points.

CI datalogging time was available for 24 patients. The overall mean CI datalogging time per day was 10.3 h (SD = 4.1; range 0–15.1). Figure 6B shows datalogging time by primary treatment motivation. There were no significant differences between the groups; however, the mean hours of device use for those desiring to restore hearing (7.8; SD = 4.4) or improve overall hearing (9.3; SD = 4.6) were well below the evidence-based datalogging recommendation of ≥12 h/day [34]. Conversely, CI datalogging exceeded the recommendation for those with primary motivations of improving speech in noise (13.2; SD = 1.7) and tinnitus (12.7; SD = 1.6). Post-CI WRS and datalogging hours by primary treatment motivation are listed in Table 3.

## 4. Discussion

In this four-year telehealth cohort of 122 adults with true SSD (mean 3-freq PTA of 26.0 dB HL in the better ear and 90.8 dB HL in the worse ear), we examined treatment motivations, initial treatment choices, and post-CI outcomes. Several key findings emerged that have relevance for patient counseling, clinical decision-making, and future research.

The most common primary reported motivations for pursuing any SSD treatment were the desire to improve overall hearing, the desire to restore hearing to the deaf side, and the desire to improve hearing in noise. Interestingly, the primary motivation did not show a statistically significant association with initial treatment choice or receiving a CI. In other words, patients’ stated reasons did not reliably predict which modality (surgical or non-surgical) they would select or ultimately receive. This could, in part, be because we asked about their treatment motivation at the start of the telehealth visit before counseling had commenced.

Several patients did choose treatment options that were misaligned with their treatment motivations. Three people with primary motivation of localization opted to proceed with HA/CROS/BiCROS instead of CI even though rerouting devices would not significantly improve localization [11,12,13,14]. A closer look at these patients revealed that CI was not recommended by the audiologist due to concerns of poor prognosis and history of VS. Similarly, while cochlear implantation has been shown to reduce tinnitus symptoms [15,18,21,22,23,24,35,36], only three patients in our study with primary motivation of tinnitus alleviation pursued CI. The majority of those who chose other treatment options stated that they did not want to undergo surgery. Interestingly, the three who received a CI proved to have the best post-CI WRSs and the longest CI wear time of any of the treatment motivation groups. Among the 26 patients whose primary motivation was improving hearing in noise, 69% chose either CROS or no treatment, despite evidence that cochlear implantation outperforms all other interventions [37]. Of note, a small percentage (5.7%) of patients cited dizziness as a primary or secondary treatment motivation for pursuing SSD treatment, but none of these patients underwent CI at our institution. Careful counseling for the SSD population should be structured and focused on the specific benefits and limitations of each treatment option to avoid unrealistic expectations and regretting their ultimate choice [5]. Overall, our findings suggest the comprehensive telehealth counseling provided by our audiologists is quite effective in assisting patients with treatment decision-making.

Similarly to what was found for the entire study cohort, CI recipients’ most common motivations for SSD treatment were to improve overall hearing (29.6%) and restore hearing to the deaf side (25.9%), both of which are known benefits of CI [15,38,39,40]. Despite the potential benefits, only 22.1% of the patients in our overall SSD cohort underwent cochlear implantation. A notable proportion (24.6%) declined due to a desire to avoid surgery, a barrier previously reported in the literature [41]. This highlights the importance of patient counseling and addressing concerns about surgical interventions in the SSD treatment journey. Insurance coverage issues also played a role in decision-making for 8.2% of patients, which remains a barrier in certain vulnerable populations like the very young and the elderly.

Amongst patients who did receive a CI, there was no significant motivation-based difference in CI datalogging time. The overall device use was 10.3 h per day, which is better than previous reports in the literature of 8 h per day [16,19,42,43] for this population. It is possible that because our center utilizes a structured shared decision-making approach in the pre-evaluation telehealth counseling appointment, those who choose to move forward with CI are more committed to CI use. Although not statistically significant, we did observe differences in CI wear time when analyzing each treatment motivation individually. Those who wished to restore hearing and/or improve overall hearing (the two primary motivations for the CI group) wore the CI for only 7.8–9.3 h per day on average, with only two (7.4%) non-users in those groups. Once the CI was implanted, patients across all other motivational backgrounds appeared willing to use the device according to the evidence-based recommendation of ≥12 h per day [34], with no non-users in these groups.

We also evaluated post-CI speech outcomes across all treatment motivations. The mean WRS of our cohort was 43.1%, which aligns with other SSD CI outcome studies [19,43,44,45,46]. When taking a closer look at each motivation, patients who underwent implantation to restore hearing to the deaf ear had significantly worse WRSs (24%) and the lowest mean datalogging time (7.8 h) of all motivations. Overall, the results point to a potential misunderstanding of the quality of hearing that a CI can realistically “restore”. This mismatch in expectations may account for the 7.4% (*n* = 2) non-use rate observed in our sample. Notably, however, this was substantially lower than the 14–36% non-use rates reported for SSD CI recipients in the literature [19,43,47]. This difference suggests that our SSD protocol, which includes pre-treatment telehealth counseling, may be an effective approach for reducing CI non-use.

While our results point to differences in CI speech outcomes and datalogging time for treatment motivations, it remains unclear who will ultimately be satisfied with the CI. A recent study from Báez Berríos and colleagues [48] reported a rate of 21% for moderate decision regret for the SSD CI population, with no direct correlation between speech outcomes and device satisfaction. This finding is similar to what has been previously reported for the general CI population [49,50,51]. Ultimately, our results suggest that CI clinicians should be particularly cautious when patients report that their main goal is to “restore hearing” in the affected ear, as realistic expectations may need to be tempered. Furthermore, patients will likely benefit from conversing with other SSD CI recipients to gain a clearer understanding of the benefits and challenges associated with this treatment option. Additionally, it is worth mentioning that even though no standardized post-CI auditory training protocol exists in the US for SSD adults [52], clinicians should encourage consistent CI-only listening practice to enhance device outcomes [53,54,55].

Our study was limited by the small number of patients who received a CI in the cohort (*n* = 27), restricting statistical power to detect differences by motivation. Future studies with a larger CI cohort could allow for robust multivariate modeling. In addition, we could not verify if patients who chose a non-surgical treatment modality during the SSD evaluation ultimately received the selected intervention, as some insurers required HA services to be provided at outside clinics. Therefore, our ability to examine relationships between treatment motivation and non-surgical treatment choices was limited.

In summary, patient-centered counseling and shared decision-making are necessary when treating SSD patients. Clinicians should inquire about the patient’s motivating factors for proceeding with a treatment option, and all treatment options should be presented comprehensively. When patients opt for cochlear implantation, realistic expectations of outcomes need to be clearly defined to avoid long-term device non-use and decision regret.

## 5. Conclusions

Collectively, these findings suggest that patient motivation, though a key consideration in shared decision-making, may not be a strong or reliable predictor of whether someone ultimately proceeds with a CI, nor of their device usage behavior. Rather, motivation may reflect initial hope or priorities but become superseded by anatomical candidacy, surgical risk tolerance, insurance, and perceived benefit during the decision process.

## Figures and Tables

**Table 1:** Demographic characteristics.

Characteristic	Mean (*SD*)	N (%)
Age (years)	56.68 (12.73)	
Sex		
Female		49 (40.2%)
Male		73 (59.8%)
Ethnicity		
Non-Hispanic		60 (49.2%)
Hispanic		52 (42.6%)
Unknown		10 (8.2%)
Race		
White		103 (84.4%)
Black/African American		9 (7.4%)
Asian		2 (1.6%)
More than one race		2 (1.6%)
Unknown		6 (4.9%)
Language		
English		95 (77.9%)
Spanish		25 (20.5%)
Russian		1 (0.8%)
Unknown		1 (0.8%)
Insurance Plan		
Private		91 (74.6%)
Medicare advantage		23 (18.9%)
Medicaid		7 (5.7%)
Self-Pay		1 (0.8%)

SD = standard deviation.

**Table 2:** Audiometric SSD characteristics.

Characteristic	N (%)	Mean (*SD*)	Range
Unaided PTA (dB HL)			
Non-Implanted Ear		97.45 (6.71)	1.67–60.67
Implanted Ear		90.75 (21.69)	41.67–120.00
Unaided WRS (%)			
Non-Implanted Ear		26.00 (12.32)	60–100
Implanted Ear		6.82 (12.13)	0–52
Pre-CI Aided WRS (%)			
Implanted Ear		8.80 (18.95)	0–77
SSD Duration		10.76 (15.78)	0.08–80.00
SSD Etiology			
Congenital	9 (7.4%)		
IV Antibiotics	1 (0.8%)		
Labyrinthitis	2 (1.6%)		
Meniere’s	13 (10.7%)		
Meningioma Surgery	2 (1.6%)		
Otologic Surgery	5 (4.1%)		
SSNHL	60 (49.2%)		
Trauma	3 (2.5%)		
Unknown	10 (8.2%)		
VS Radiation	4 (3.3%)		
VS Surgery	9 (7.4%)		

PTA = 3-frequency pure tone average (500, 1000, 2000 Hz); WRS = word recognition score; HL = hearing level; CI = cochlear implant; SSD = single-sided deafness; IV = intravenous; SSNHL = sudden sensorineural hearing loss; VS = vestibular schwannoma; SD = standard deviation.

**Table 3:** Single means for post-CI word recognition score and datalogging for primary treatment motivation.

Primary Treatment Motivation	CI Word Recognition Score				CI Datalogging Hours per Day			
	Mean (%)	*SD*	Median	*n*	Mean (h)	*SD*	Median	*n*
Hearing Restoration	24	22.5	30	7	7.8	4.4	8.9	7
Overall Hearing	52.3	17.1	53	6	9.3	4.6	9.7	7
Localization	25.3	28.4	20	3	11.6	17.1	12.6	3
Speech in Noise	55.3	14.7	50	3	13.2	1.7	14	3
Tinnitus	58.7	28.4	64	3	12.7	1.6	12.7	2
Unknown	92	0	92	1	14.3	1.2	14.3	2

CI = cochlear implant; *n* = sample size; *SD* = standard deviation; h = hours.

## Data Availability

The raw data supporting the conclusions of this article will be made available by the authors on request.

## Abbreviations

SSD	Single-Sided Deafness
CROS	Contralateral Routing of Signal
BiCROS	Bilateral Contralateral Routing of Signal
BCD	Bone Conduction Device
HA	Hearing Aid
CI	Cochlear Implant
US	United States
WRS	Word Recognition Score
CNC	Consonant Nucleus Consonant
SSNHL	Sudden Sensorineural Hearing Loss
VS	Vestibular Schwannoma
